# Opioid-Induced Nausea and Vomiting in Patients With Cancer: A Narrative Review

**DOI:** 10.7759/cureus.108798

**Published:** 2026-05-13

**Authors:** Daniela M Perilla Orozco, Adalberto D Pantoja Molina, Juan P Valencia Quivano, Carlos E Cabrera Velasco, Laura N Ricaurte Gracia, Jorge A Sánchez-Duque

**Affiliations:** 1 Pain Medicine and Palliative Care Unit, Universidad Militar Nueva Granada, Instituto Nacional de Cancerología, Bogotá, COL; 2 Family Medicine Unit, Department of Social Medicine and Family Health, Universidad del Cauca, Popayán, COL; 3 Gastroenterology Unit, Universidad del Rosario, Bogotá, COL; 4 Pain Medicine and Palliative Care Unit, Fundación Universitaria de Ciencias de la Salud, Hospital Universitario San José, Bogotá, COL; 5 Geriatrics Unit, Universidad Nacional de Colombia, Bogotá, COL

**Keywords:** antiemetics, cancer patients, nausea and vomiting, opioid-induced nausea and vomiting, opioid medication, palliative care

## Abstract

Opioid-induced nausea and vomiting (OINV) represent a frequent and clinically significant complication in patients with cancer. They negatively affect adherence to analgesic therapy, pain control, and overall quality of life. Their pathophysiology is multifactorial and involves activation of the chemoreceptor trigger zone through dopaminergic and serotonergic pathways, impairment of gastrointestinal motility leading to delayed gastric emptying and reduced peristalsis, and vestibular modulation mediated by histaminergic and muscarinic pathways. These mechanisms are further influenced by individual susceptibility and pharmacological factors. Despite their clinical relevance, international guidelines primarily focus on chemotherapy- or radiotherapy-induced emesis, leaving a gap in the specific management of OINV. This narrative review synthesizes the available evidence and proposes a structured diagnostic and therapeutic approach tailored to this condition. A stepwise management model is presented, integrating non-pharmacological strategies and sequential pharmacological treatment according to the predominant pathophysiological mechanism. Explicit criteria for therapeutic failure and referral to specialized care are also included. This approach aims to optimize symptom control, support rational opioid rotation when indicated, and improve quality of life in patients with cancer receiving palliative care.

## Introduction and background

Nausea and vomiting are among the most common and distressing symptoms in cancer patients, especially those with advanced disease [1]. These symptoms signal marked declines in quality of life, physical ability, and well-being for both patients and families [1,2]. Their causes are complex [2,3]. Tumor location, nervous system or digestive tract involvement, and metabolic disruptions all play roles [3,4]. Rates are highest in brain, esophageal, stomach, and pancreatic cancers [3-5]. These symptoms arise not only from tumor biology but also from treatments used in managing the disease [6-8].

Chemotherapy is a common cause of vomiting [7,8]. It occurs when chemotherapy drugs act on central and peripheral emetogenic pathways [9-11]. Outside of chemo-radiotherapy, nausea and vomiting can result from overlapping causes [3,5,12]. These include altered gut-brain signaling, tumor blockage or invasion, medication-related delayed gastric emptying, and metabolic abnormalities, especially in advanced disease [1,3,10]. Other factors include neurological disorders, emotional stress, and drug interactions [10,11]. These causes can lead to different responses to anti-nausea treatment among individuals [13,14].

In advanced cancer, opioids are the cornerstone for managing moderate to severe pain and refractory dyspnea, significantly improving symptom control and patient comfort [6,12]. However, opioid-induced nausea and vomiting (OINV) may appear shortly after opioid initiation or dose escalation and can limit analgesic adherence, interfere with dose titration, and worsen symptom burden [6,12,15,16]. These effects account for the early onset of symptoms after opioid initiation and the variability in clinical presentation across opioid agents and formulations [7,15,17].

The incidence of OINV in cancer patients ranges from 15% to 40% [7,8]. Nausea affects 25-31%, while vomiting occurs in 17-24% [16,17]. These rates vary with opioid type, dose, route, and titration speed [7,8,17,18]. Codeine, morphine, and oxycodone have a higher emetogenic burden. Transdermal fentanyl and buprenorphine are generally better tolerated in some patients due to stable plasma levels and fewer peak-related side effects, but evidence does not conclusively prove lower emetogenic potential across opioids [2,19,20]. These drug differences stress the need for opioid-specific risk stratification in OINV management [6,8,12].

Despite its high prevalence and its negative impact on analgesic adherence and quality of life, there are no internationally accepted guidelines that systematically direct the diagnostic and therapeutic approach to nausea and vomiting outside the context of chemotherapy or radiotherapy [9,19,21]. Current recommendations from the Multinational Association of Supportive Care in Cancer (MASCC), the European Society for Medical Oncology (ESMO), the National Comprehensive Cancer Network (NCCN), and the American Society of Clinical Oncology (ASCO) focus predominantly on emesis associated with antineoplastic treatments, thereby leaving a clinically significant gap in the management of OINV [9,19,22].

This article addresses this unmet need by presenting a narrative synthesis of current evidence and introducing a structured clinical framework for diagnosing and managing OINV in cancer patients. This framework merges opioid-related risk stratification with a stepwise therapeutic model, aiming to optimize decision-making, improve symptom control, preserve analgesic effectiveness, reduce unnecessary polypharmacy, and elevate quality of life in palliative care [6,9,12].

## Review

Methodology

A narrative review of the literature was conducted without statistical analysis. A multidisciplinary team performed the review. Specialists in palliative care, anesthesiology, gastroenterology, internal medicine, geriatrics, family medicine, and epidemiology contributed their expertise. The team analyzed the evidence qualitatively. This collaboration maintained clinical relevance and clear methods.

Information Search

A comprehensive bibliographic search was performed across six electronic databases: PubMed, Scopus, ScienceDirect, SciELO, Redalyc, and Google Scholar. Publications from 2006 to 2025 were included. English MeSH terms like "nausea", "vomiting", "antiemetics", "cancer", and "palliative care" were used. Search terms were combined with field tags, proximity, and Boolean operators to improve sensitivity and specificity. Eligible studies were (1) reviews, (2) clinical practice guidelines, (3) interventional trials, and (4) observational studies (cross-sectional, case-control, cohort). Articles had to be full-text in English or Spanish. Exclusion criteria were letters to the editor, book chapters, and duplicates.

Selection, Appraisal, and Synthesis of Evidence

Two reviewers independently screened all records, assessing their relevance and quality for the review. Articles approved by both were included, and discrepancies were resolved by a third author. Data were extracted and organized into thematic domains: epidemiology, pathophysiology, diagnostics, risk factors, prevention, treatment, and referral. Data extraction and verification were supervised by a faculty member from the Palliative Care Group at Instituto Nacional de Cancerología (Colombia) and the epidemiology lead, ensuring consistency and clinical applicability.

Analysis and Formulation of Recommendations

The interdisciplinary team discussed findings collaboratively and employed an evidence-informed, deliberative process to contextualize the data within clinical practice. They formulated structured, stepwise clinical recommendations to guide multidisciplinary OINV management in cancer patients, drawing on both synthesized evidence and institutional experience. Additional references were incorporated during manuscript preparation to address emerging clinical questions and enhance conceptual coherence.

Ethical Considerations

This study is a narrative review based on previously published literature. It did not involve direct interaction with human participants or use identifiable personal data. The review followed the ethical principles outlined in the Declaration of Helsinki. It also respected international guidelines for research using secondary data sources.

Results

Evidence Synthesis

Epidemiology: Nausea and vomiting are highly prevalent in advanced cancer patients in palliative care. The frequency increases as the disease progresses and death approaches [14]. In specialized programs, the prevalence of nausea is about 36% at first clinical contact. It rises to 62% one to two months before death. During the final week of life, it exceeds 70%. This pattern reflects a substantial, time-dependent symptom burden closely linked to disease progression [14].

Within the broad etiological spectrum of these symptoms, opioids represent one of the main pharmacological triggers of nausea and vomiting unrelated to specific oncological treatments [23,24]. OINV is defined by its temporal association with opioid initiation or dose titration, after excluding alternative predominant causes. Its true magnitude is likely underestimated due to the coexistence of multiple pathophysiological mechanisms in advanced cancer [8]. Opioid-induced emesis has been reported in more than 60% of patients beginning these agents. Nausea predominates over vomiting. A higher frequency is observed during the initial phases of treatment before pharmacological tolerance develops [14].

From an epidemiological perspective, nausea associated with opioid use occurs in approximately 25% to 40% of patients with cancer, whereas vomiting is observed in roughly 15% to 25%, with considerable variability across studies and care settings [8]. These symptoms are most frequently reported during the first days following opioid initiation or dose escalation; however, they may persist in a clinically meaningful proportion of patients and are associated with impaired quality of life and reduced analgesic adherence, including dose reduction or treatment discontinuation [8]. In resource-limited settings, the burden of opioid-related adverse events, including OINV, remains substantial, suggesting that its epidemiological magnitude is influenced not only by the pharmacological agent but also by health system context and supportive care capacity [25].

Heterogeneity in the presentation of OINV has been confirmed through population-based analyses, indicating that its distribution is influenced not only by the specific opioid prescribed but also by individual susceptibility factors [17]. In a multicenter retrospective cohort study of 416 patients with cancer-related pain receiving opioids, the overall incidence of OINV was 18.9%, with similar estimates in the derivation cohort (17.7%) and the validation cohort (22.0%), suggesting a consistent population-level burden of the event [17]. Multivariable analysis identified five independent predictors of OINV: a history of motion sickness, nocturnal sleep duration of fewer than five hours, non-initial opioid use, recent dose adjustments, and prior chemotherapy-induced nausea and vomiting, with the latter showing the strongest association [17]. Conversely, variables such as age, sex, tumor type, or specific opioid agent did not demonstrate independent associations [17].

Based on these determinants, a predictive nomogram was developed with adequate discriminative performance, demonstrating a concordance index of 0.835 in the training cohort and 0.810 in the validation cohort, enabling individual risk estimation and stratification of high-risk populations before or during opioid exposure [17]. Collectively, these findings confirm that OINV represents a predictable and non-random adverse event whose population burden is modulated by identifiable clinical factors, thereby providing the epidemiological framework necessary to interpret the variability observed in the prevalence of nausea and vomiting across different opioid regimens [17].

In this context, and in the absence of internationally endorsed guidelines specifically addressing the management of OINV, Table 1 summarizes the reported prevalence of nausea and vomiting associated with the principal opioids available in Colombia, providing a pragmatic epidemiological reference to support emetogenic risk assessment and individualized analgesic selection in clinical practice [6,8,19].

**Table 1:** Reported prevalence of nausea and vomiting by opioid agent in patients with cancer. Source: Developed by the authors based on references [26-33].

Authors	Opioid agent	Nausea (%)	Vomiting (%)
Besic et al. (2020) [26]	Tramadol	36%	9%
Vadivelu et al. (2013) [27]	Tapentadol	30%	18%
Campora et al. (1991) [28]	Morphine	18.3%	28%
Li et al. (2021) [29]	Hydromorphone	13%	18%
Ma et al. (2016) [30]	Oxycodone	20.8%	17.2%
Wang et al. (2005) [31]	Codeine	19.7%	6.5%
Rodríguez et al. (2007) [32]	Hydrocodone	13.3%	6.7%
Chwistek et al. (2023) [33]	Buprenorphine	13%	9%
Yu et al. (2005) [20]	Fentanyl	13.6%	3.9%

Pathophysiology of Nausea and Vomiting

Central mechanisms: Nausea and vomiting are closely related but physiologically distinct phenomena that involve partially overlapping yet non-identical neural circuits [3,4]. Nausea is defined as a subjective visceral sensation characterized by discomfort and the urge to vomit, whereas vomiting constitutes an objective motor event resulting from a coordinated neuromuscular reflex culminating in expulsion of gastric contents [2,5,34]. Both processes depend on an integrated network of peripheral and central signaling pathways designed to respond to potentially harmful chemical, mechanical, infectious, or sensory stimuli [3,5].

From a neuroanatomical perspective, afferent input arises primarily from the gastrointestinal tract through the enteric plexuses and vagal pathways, as well as from humoral stimuli that directly access the central nervous system [3,4]. These signals converge within the brainstem, particularly at the nucleus tractus solitarius and the chemoreceptor trigger zone located in the area postrema, a circumventricular structure lacking an effective blood-brain barrier and functioning as a sensor of circulating toxins [3,11]. Within these integrative centers, multiple neurotransmitters, including serotonin, dopamine, substance P, and histamine, modulate both the perception of nausea and the activation of the emetic reflex through complex receptor-mediated interactions [3,11,19].

Peripheral mechanisms: Peripheral afferent input arises primarily from the gastrointestinal tract through the enteric plexuses and vagal pathways, as well as from humoral stimuli that directly access central emetic structures [3,4]. Gastrointestinal irritation, distension, delayed gastric emptying, impaired intestinal transit, and visceral inflammation may activate vagal and splanchnic afferents that project to brainstem integrative centers, thereby contributing to nausea and vomiting through peripheral-central signaling [3-5,11].

The motor response underlying vomiting involves a highly coordinated sequence that engages the diaphragm, abdominal musculature, glottis, and lower esophageal sphincter under medullary control [5,34]. Concurrently, activation of autonomic responses, such as hypersalivation, diaphoresis, pallor, and peripheral vasodilation, frequently accompanies nausea, reflecting its close association with autonomic nervous system regulation [4,14].

Cancer-related sensitization of the emetic system: In patients with cancer, nausea and vomiting occurring outside the context of chemotherapy or radiotherapy develop upon a vulnerable emetic system characterized by reduced physiological reserve and heightened reactivity to emetogenic stimuli [1,11]. Rather than reflecting an isolated mechanism, these symptoms represent a process of secondary sensitization driven by structural, metabolic, and neurobiological alterations associated with advanced disease [3,5,35].

The gut-brain axis plays an integrative role by connecting visceral signals to the area postrema, the nucleus tractus solitarius, the insular cortex, and limbic structures involved in the emotional and cognitive perception of nausea [2,11,35]. Tumor infiltration, partial obstruction, gastroparesis, and peritoneal involvement increase visceral and autonomic afferent activation, while common metabolic disturbances, such as hypercalcemia, uremia, or hepatic failure, may directly stimulate central emetic centers through neurohumoral pathways [4,5,11,15].

Additional contributors include systemic inflammation, malnutrition, and cancer cachexia, which impair gastrointestinal motility and increase visceral hypersensitivity, thereby amplifying afferent signaling toward brainstem integrative centers [2,35]. Within this sensitized neurobiological milieu, stimuli that would ordinarily remain subthreshold may precipitate persistent or refractory symptoms, explaining the substantial clinical burden and the marked heterogeneity in therapeutic response observed in advanced cancer [5,12].

Opioid-specific mechanisms: Within the context of an emetic system sensitized by the synergistic interaction of multiple mechanisms that lower the emetic threshold and facilitate symptom expression, opioid therapy stimulates μ-opioid receptors in the chemoreceptor trigger zone of the area postrema, a structure particularly vulnerable due to the absence of an effective blood-brain barrier, thereby promoting secondary activation of dopaminergic and serotonergic pathways implicated in the emetic response [12,23,34,35]. This mechanism explains the early onset of nausea following opioid initiation or titration and its temporal association with recent dose adjustments [7,17,18].

Concurrently, opioids inhibit gastrointestinal motility, resulting in delayed gastric emptying and reduced intestinal transit, thereby promoting luminal distension and activation of vagal afferents that project to central emetic centers [6,11,17]. OINV should therefore be conceptualized as a pharmacologically induced decompensation occurring within a previously vulnerable emetic system rather than as an isolated adverse event [15,23,34].

This pathophysiological framework supports the systematic evaluation of opioid type, dosage, route of administration, and titration rate, as well as the integration of therapeutic strategies aimed not only at symptomatic control but also at correcting the precipitating factor and restoring functional balance within the emetic network [6,8,12].

Diagnostic Approach

Given the multifactorial causes of nausea and vomiting in patients with cancer, their evaluation requires a structured diagnostic approach aimed at identifying the primary cause, establishing symptom progression, recognizing contributing comorbidities, and understanding the overall clinical condition of the patient [9]. In clinical practice, these symptoms usually occur in three main scenarios: exposure to chemotherapy and/or radiotherapy, tumor progression without active oncological treatment, and opioid use for pain management [2-5,15].

In patients receiving chemotherapy, the initial assessment should evaluate the emetogenic potential of the treatment plan, especially in protocols that include highly emetogenic agents such as cisplatin, anthracyclines, dacarbazine, or carmustine [9,10]. It is also crucial to assess symptom timing, whether acute, delayed, or anticipatory, and adherence to antiemetic prophylaxis, particularly in cases of breakthrough emesis where preventive strategies may need adjustment [9,10,36,37]. Since chemotherapy- and radiotherapy-induced nausea and vomiting are managed by well-established international guidelines, this review emphasizes non-antineoplastic causes, a field where important diagnostic and therapeutic gaps still exist [13,15,38].

In patients with progressive cancer who are not undergoing active oncological treatment, evaluation should be comprehensive and aimed at the careful exclusion of structural, functional, and metabolic causes related to advanced disease [2,5,6]. Priority should be placed on identifying partial or complete intestinal obstruction, gastroparesis, central neurological involvement, including brain or meningeal metastases and increased intracranial pressure, peritoneal or hepatic involvement, and systemic disturbances such as hypercalcemia, uremia, or hepatic failure [4,5,15]. These conditions may coexist and act together on both central and peripheral pathways of the emetic reflex [11,35].

Within this clinical context, opioids, cornerstone agents in the management of cancer-related pain, represent a frequent and clinically significant cause of nausea and vomiting through direct stimulation of the chemoreceptor trigger zone and inhibition of gastrointestinal motility [7,17]. OINV typically manifests within the first 24 to 72 hours after treatment initiation or dose escalation [7,17]. When symptoms persist beyond five to seven days, careful reassessment of the analgesic prescription is warranted, including dose modification, route adjustment, or rotation to opioids with a more favorable emetogenic profile, as part of an integrated diagnostic and therapeutic strategy [6,7].

Differential Diagnosis

Nausea and vomiting in patients with cancer have a multifactorial cause that extends beyond traditional oncological reasons, including chemotherapy, radiotherapy, tumor progression, or opioid use, therefore requiring systematic evaluation of conditions not directly related to the cancer, especially in patients with significant comorbidities or advanced organ dysfunction [12,39]. A comprehensive diagnostic approach minimizes clinical errors, helps identify potentially reversible conditions, and recognizes the frequent coexistence of central and peripheral mechanisms, with symptom timing playing a key role in guiding personalized diagnostic and treatment choices [12,39].

In acute presentations, the differential diagnosis should include infectious causes, adverse drug reactions, including opioids, antiarrhythmics, antihypertensives, diuretics, and oral antidiabetic agents, and surgical entities such as intestinal obstruction [16,23]. Neurological causes, including migraine and vestibular vertigo, cardiovascular conditions such as acute coronary syndrome, and disorders associated with raised intracranial pressure must also be considered in the appropriate clinical context [16,34,40]. Concurrently, severe metabolic disturbances, including diabetic ketoacidosis, adrenal insufficiency, uremia, or hepatic encephalopathy, should be actively excluded, as they may induce emesis through toxic or neurohumoral mechanisms [11,16,23].

**Figure 1:**
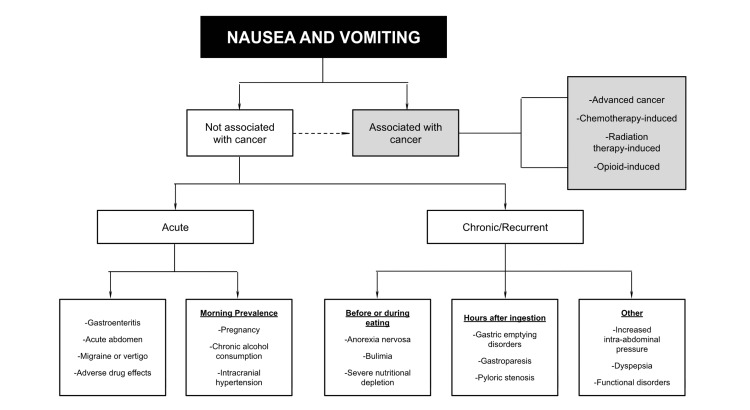
Algorithm for the differential diagnosis of nausea and vomiting in patients with cancer. The algorithm differentiates causes directly related to the underlying malignancy and its treatments (chemotherapy, radiotherapy, and opioids) from non-cancer-related conditions, classified according to clinical presentation as acute or chronic/recurrent, in order to guide systematic etiological evaluation and rational therapeutic decision-making. Source: Developed by the authors using Microsoft Word (Microsoft Corporation, Redmond, WA) based on references [1-7].

In chronic cases, the timing of symptoms relative to food intake is a key diagnostic factor [4,5]. Immediate or anticipatory nausea indicates dominance of central neurochemical mechanisms, whereas delayed symptoms associated with early satiety suggest impaired gastric emptying, such as gastroparesis or pyloric stenosis [4,5]. In patients with advanced cancer and cachexia, severe malnutrition affects appetite regulation, slows gastric motility, and heightens visceral sensitivity, resulting in a complex clinical spectrum with overlapping functional and neurological features [11,24,41]. Other conditions to consider include those associated with increased intra-abdominal pressure, functional dyspepsia, and cyclic vomiting syndrome [42-44]. The differential diagnostic algorithm presented in Figure 1 provides a structured framework to enhance clinical precision in this setting [1-5].

Tools for the Assessment of OINV

Nausea and vomiting are inherently subjective symptoms, and their clinical assessment presents a significant challenge in oncology and palliative care, particularly given interindividual variability and functional impact [13,19]. In the specific context of OINV, no diagnostic scale has been developed solely for this condition; however, several validated tools exist to quantify symptom severity, support treatment decisions, and monitor response to therapy [13,19,45]. Systematic use of these instruments enables early detection of clinically significant OINV, optimizes medication adjustments, and helps prevent complications such as dehydration, malnutrition, or discontinuation of analgesics [4,25,35,45]. Among these tools, three scales have shown clinical usefulness across various care settings and palliative situations [14,35].

The Index of Nausea, Vomiting, and Retching (INVR), also known as the Rhodes Index, is a self-report tool designed to assess the multidimensional emetic experience, including nausea, vomiting, and retching, as well as their frequency, duration, and associated distress [14]. Although its primary use has been in chemotherapy-induced and postoperative emesis, its detailed nature makes it especially useful for baseline assessment and follow-up of OINV in patients with cancer [46]. The INVR uses Likert-style items scored from 0 to 4 and is recommended as a retrospective record every 12 hours, based on clinical agreement between the patient and the care team [47,48]. Interpreting the total score requires careful attention to reverse-coded items, which is crucial for accurately assessing overall severity [49]. Based on the total score, severity can be categorized as absent, mild (<8 points), moderate (9-16), severe (17-24), or very severe (>24), allowing for meaningful clinical stratification to guide therapy decisions [50]. During follow-up, an adequate response is defined as at least a one-category reduction from baseline severity, while persistent severe scores or lack of improvement indicate an inadequate response and the need for further therapeutic adjustments [45,50].

The MASCC Antiemesis Tool (MAT) was created by the Multinational Association of Supportive Care in Cancer (MASCC) as a standardized instrument for evaluating chemotherapy-induced nausea and vomiting, with international validation and wide use in clinical oncology [51]. The tool has been further adapted for use in OINV, especially as an early monitoring tool following opioid exposure [52]. The MAT includes eight items that assess the presence, frequency, and intensity of nausea and vomiting and is usually used as a serial diary during the initial days of treatment [51,52]. Unlike cumulative scoring systems, the MAT provides a clinical control profile rather than a total numerical score, with ≥4 vomiting episodes or nausea intensity ≥6 on a numerical scale considered clinically significant [52]. Its use is particularly important during the first three to five days after starting opioids or adjusting the dose, the period when OINV most often appears; symptoms that persist beyond one week should lead to reevaluation of the cause and consideration of opioid rotation or medication adjustment [7,17,52].

The Edmonton Symptom Assessment System-Revised (ESAS-r) is a widely validated palliative care tool for quick screening and ongoing monitoring of overall symptom burden, including nausea, using numerical scales from 0 to 10 [53]. Its usefulness in OINV is in identifying clinical imbalances, especially when nausea scores rise despite adequate pain control, signaling a need for targeted evaluation [53]. Symptom severity is categorized as mild (1-3), moderate (4-6), or severe (7-10), with nausea item scores of 4 or higher considered clinically significant [53]. The ESAS-r is designed for repeated assessments, usually daily, and an adequate response is defined as a decrease of at least three points from the baseline; smaller reductions or continued high scores suggest an insufficient response and indicate the need to optimize antiemetic or analgesic treatment [45,53].

Risk Factors and Prevention

In patients with cancer, nausea and vomiting are linked to negative effects on physical, emotional, and nutritional health, as well as decreased adherence to treatment plans, including pain management, thereby leading to functional decline and greater suffering [15,21]. Early identification of individual risk factors is essential, as recent palliative care reviews confirm that opioid-related adverse effects, particularly nausea and vomiting, remain major contributors to treatment intolerance, opioid rotation, and reduced adherence to analgesic therapy in patients with advanced cancer [13,19,25,54]. The most consistently reported risk factors include a personal history of vomiting, female sex, younger age, anxiety, sleep disturbances, and previous exposure to highly emetogenic treatments such as chemotherapy​​​​​ [9,10,45]. In patients with these traits, starting or adjusting opioids may raise the risk of OINV, making recognition of these factors important for preventive decision-making [13,19].

In individuals with high baseline risk or documented prior OINV, initiating antiemetic prophylaxis when starting opioids with higher emetogenic potential has been suggested as a way to reduce early symptom severity [7,18,37]. However, this method remains debated, as most OINV episodes are temporary, self-resolving, and can be managed with opioid adjustments or targeted symptomatic treatment without the need for extended prophylaxis [7,17,37]. When prophylaxis is appropriate, it should be tailored to the individual and limited in duration, usually lasting three to five days, covering the initial high-risk period after starting or increasing opioid doses, to prevent unnecessary polypharmacy and related side effects [7,17,24].

Non-pharmacological interventions can be a useful addition, especially for patients with anticipatory symptoms, significant anxiety, or high emotional distress related to their symptoms [13,43]. Educating patients and caregivers about the emetogenic risks of opioids, normal symptom patterns, and their often-transient nature, along with relaxation techniques and psychoeducational support, may help reduce the perceived severity of nausea [2,13]. However, the evidence supporting these approaches is limited and varied, so they should be considered as complementary strategies within a broader multimodal treatment plan [13,42,43]. Overall, preventing OINV should be viewed as a selective, risk-based, and stepwise process focused on reducing symptoms while avoiding overmedicalization and unnecessary polypharmacy [13-15].

Treatment

Management of nausea and vomiting in cancer patients should be individualized, stepwise, and guided by the main cause, with particular focus on identifying cases caused by OINV due to their high prevalence and potential effect on pain management and quality of life [15,39]. The diagnosis of OINV is mainly made through the timing between starting or adjusting opioids, including changes in dose, formulation, or route, and the beginning of symptoms. This step is crucial before increasing or intensifying antiemetic treatment [6,17].

Early recognition of the opioid’s causal role enables the optimization of the analgesic regimen, reduces unnecessary polypharmacy, and improves overall treatment tolerability, especially in patients with advanced disease and limited physiological reserve [19,36]. Before starting or increasing antiemetic therapy, a systematic review of the analgesic approach is recommended, including assessment of titration speed, total daily dose, route of administration, presence of opioid-induced constipation, and hydration status, all of which directly affect the pathophysiology of OINV [7,17]. In cases of persistent or difficult-to-control OINV, early consideration should be given to rotating opioids toward agents with a lower emetogenic profile, as well as modifying the route of administration when clinically appropriate, as part of an integrated and patient-centered therapeutic strategy [7,17].

Non-pharmacological Management

Along with etiological assessment and pharmacological treatment, general supportive measures should be implemented, as they form a fundamental part of management regardless of symptom severity [13,42,43]. These measures include patient and caregiver education, environmental control, and the optimization of nutritional and emotional well-being, aiming to reduce activation of sensory, autonomic, and vestibular pathways involved in the emetic reflex [42,43].

From a nutritional perspective, dividing food intake into small portions, prioritizing cold or room-temperature meals, and avoiding fatty, spicy, or strong-smelling preparations are recommended to reduce olfactory stimulation and vagal activation [2,13,54]. In clinical practice, foods such as jelly, chilled yogurt, smoothies, or soft purées are often better tolerated and facilitate gradual caloric intake without worsening symptoms [2,5]. Environmental control, by decreasing strong odors, loud noises, and excessive head movements, may reduce vestibular stimulation and improve oral tolerance [13,24].

In selected patients, complementary interventions such as acupuncture, especially stimulation of the P6 (Neiguan) point, or aromatherapy with ginger, lavender, or lemon may be integrated into a multimodal approach, recognizing that their benefits are modest and that the supporting evidence remains limited and inconsistent [42,54].

Pharmacological Treatment

Pharmacological therapy is a key part of both preventing and treating nausea and vomiting in cancer patients [22,37]. Its use is especially important for those exposed to highly emetogenic agents and when non-drug approaches are not enough [19,36]. The National Comprehensive Cancer Network (NCCN) released its updated 2025 guidelines for chemotherapy- and radiotherapy-induced emesis, offering clear algorithms for these cases [21]. However, since these guidelines do not specifically address situations unrelated to cancer treatments, this manuscript proposes an alternative, mechanism-based approach grounded in the best evidence and rooted in pharmacodynamic principles [12].

**Figure 2:**
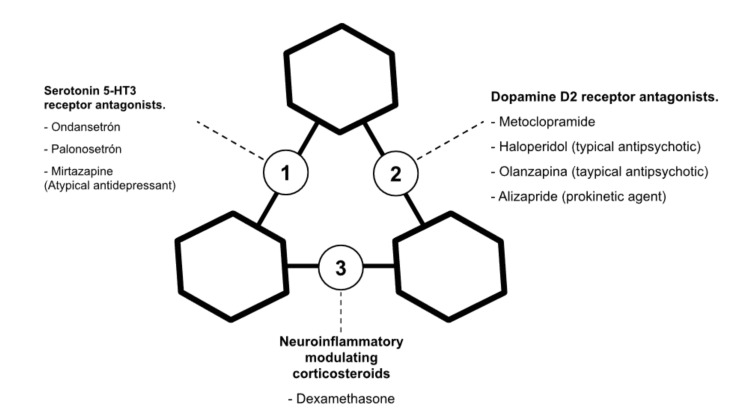
Available pharmacological agents for the management of opioid-induced nausea and vomiting (OINV) in patients with cancer. Neurokinin-1 receptor antagonists (NK₁-RAs: aprepitant and fosaprepitant) are also approved antiemetic agents in Colombia; however, their use is primarily reserved for the prophylaxis and treatment of highly emetogenic chemotherapy- or radiotherapy-induced emesis, in accordance with Multinational Association of Supportive Care in Cancer (MASCC)/European Society for Medical Oncology (ESMO) and National Comprehensive Cancer Network (NCCN) 2025 guidelines [9], and are therefore not represented in the main schematic. Source: Developed by the authors using Microsoft Word (Microsoft Corporation, Redmond, WA) based on references [1-7,21].

Selection of the antiemetic agent should be personalized based on the main cause and patient comorbidities, as these factors greatly affect both treatment safety and effectiveness [21]. In the context of OINV, receptor-targeted therapy, especially agents that influence dopaminergic, serotonergic, and neurokinin pathways, should correspond with the underlying pathophysiological mechanism and the clinical presentation of symptoms [12,19]. The principal pharmacological agents employed in the management of OINV are summarized in Figure 2.

Selection of antiemetic therapy in patients with OINV should be etiological, sequential, and context-sensitive, considering the pharmacological profile of the opioid, the predominant pathophysiological mechanism, and associated comorbidities [6,39]. Before escalating antiemetic therapy, conditions that may affect safety or treatment efficacy must be systematically excluded [2,23]. In the presence of complete malignant bowel obstruction, prokinetic agents are contraindicated, although they may be considered in confirmed cases of pseudo-obstruction [1,15]. In patients with Parkinson’s disease or extrapyramidal disorders, dopamine antagonists should be avoided due to the risk of neurological worsening [2]. Similarly, in the presence of QTc prolongation or other cardiac conduction abnormalities, 5-HT3 antagonists should be used with caution [10,21]. When the oral route is not feasible, due to intractable vomiting, acute abdomen, or raised intracranial pressure, subcutaneous or parenteral administration ensures therapeutic continuity and timely symptom control [39,41].

5-HT3 receptor antagonists are an effective antiemetic option in various oncological settings, and their concomitant use with agents that share the same mechanism should be avoided to prevent redundant therapy and increased side effects [21]. In OINV, they may be considered as an initial choice for patients experiencing predominant nausea without significant constipation [6,8]. Ondansetron has shown clinical effectiveness in controlling vomiting; however, it may worsen opioid-induced constipation and, in some cases, increase gastrointestinal symptoms [1,22,55]. Therefore, its use should be cautious and limited in duration, ideally not exceeding seven days, due to the risk of severe constipation and QTc prolongation [9,21].

In patients with slowed intestinal transit or high risk of opioid-induced constipation, prolonged ondansetron use may be counterproductive, requiring early reassessment and switching to agents with alternative mechanisms of action [9,36]. Palonosetron, a second-generation antagonist with higher 5-HT3 receptor affinity and a longer half-life, may be considered in specific cases of persistent emesis, although limited availability and increased cost restrict its routine clinical use [56,57]. Mirtazapine, due to its multi-receptor profile (5-HT2, 5-HT3, H1, and α2 antagonism), serves as a useful alternative in persistent OINV associated with anorexia, insomnia, or mood symptoms, offering additional antiemetic benefits in patients with functional decline or intolerance to other agents [36,58,59].

Dopamine antagonists are a key treatment for OINV, especially when there is impaired gastrointestinal motility or opioid-induced constipation coexists [12]. In this case, metoclopramide may be considered a first-line option in mild OINV, particularly in patients at high risk of constipation, due to its prokinetic effects that help reverse opioid-induced delayed transit and avoid the constipating effects of 5-HT3 antagonists [11,22]. This approach is reasonable when nausea is the main symptom, with no intractable vomiting, and no neurological contraindications [25-27].

In refractory OINV or when central mechanisms are suspected, low-dose haloperidol (0.5-1 mg every 8-12 hours) is an effective option, with careful monitoring for extrapyramidal adverse effects [5,6]. Alizapride may be considered in frail patients or older adults due to its more favorable tolerability profile [9,10,21]. Olanzapine, which has potent central dopaminergic blockade and a broad multi-receptor profile, emerges as a viable alternative in refractory cases, especially when anxiety, insomnia, or anorexia coexist [60,61]. Corticosteroids, particularly dexamethasone, should be reserved as third-line therapy in refractory OINV, especially when an inflammatory component, tumor infiltration, vasogenic edema, or abdominal distension is present [22,35,62]. Short treatment courses are recommended, with titration according to clinical response and gradual tapering after improvement, while closely monitoring for metabolic, neuropsychiatric, and infectious adverse effects, as well as potential drug interactions [3,22,35]. The recommended prescribing regimens for the most commonly used agents in Colombia are detailed in Table 2 [3,22].

**Table 2:** Pharmacological agents and formulations available in Colombia for the management of opioid-induced nausea and vomiting. * ADD: administered as a single daily dose; ** BDD: divided into two doses (every 12 hours); *** TDD: divided into three doses (every eight hours); **** SD: single dose. Source: Developed by the authors based on references [1-10].

Mechanism of action/pharmacological class	Drug/formulation	Initial dose	Maintenance dose	Maximum dose	Treatment duration
5-HT3 receptor antagonists	Ondansetron - Tablets: 4 mg, 8 mg. Ampoules (injection): 4 mg/2 mL, 8 mg/4 mL. Palonosetron ampoule (injection): 250 micrograms/5 mL	Intravenous: 8-16 mg. Oral: 16-24 mg total daily dose (TDD)***. Preferably administered 30 minutes prior to chemotherapy. Intravenous: 250 micrograms (single dose). Preferably administered 30 minutes prior to chemotherapy	24 mg TDD***	32 mg TDD***, 250 mcg ADD*	≤ 7 days, ≤ 7 days
Prokinetic agents/dopamine D2 antagonists	Metoclopramide - Tablets: 10 mg. Oral solution (drops): 4 mg/mL (30 mL bottle). Ampoules (injection): 10 mg/2 mL	10-20 mg BDD**	30-40 mg BDD**	40-60 mg TDD***	≤ 7 days
Typical antipsychotics	Haloperidol - Tablets: 5 mg, 10 mg. Oral solution (drops): 2 mg/mL (15 mL, 20 mL, and 30 mL bottles). Ampoules (injection): 5 mg/mL	IV dose: 1 mg ADD*. Oral dose: 2.5-5 mg ADD*	2 mg BDD**, 10 mg BDD**	3 mg TDD***, 15 mg TDD***	≤ 7 days
Atypical antipsychotics	Olanzapine - Tablets: 5 mg, 10 mg	2.5-5 mg ADD*	5 mg ADD*	20 mg BDD*	≤ 7 days
Corticosteroids	Dexamethasone - Tablets: 4 mg. Oral solution: 1 mg/5 mL. Ampoules (injection): 4 mg/mL; 8 mg/2 mL	4 mg ADD*	4 mg ADD*	8 mg ADD*	2-5 days
Atypical antidepressants	Mirtazapine - Tablets: 15 mg, 30 mg	7.5-15 mg ADD*	7.5-15 mg ADD*	30 mg ADD*	≤ 7 days
Prokinetic agents/dopamine D2 antagonists	Alizapride - Oral solution (drops): 12 mg/mL. Tablets: 50 mg. Ampoules (injection): 50 mg/2 mL	100 mg BDD**	150 mg TDD***	200 mg TDD**	≤ 7 days
Neurokinin-1 (NK1) receptor antagonists	Aprepitant - Capsules: 80 mg, 125 mg	125 mg. Preferably administered 1 hour prior to chemotherapy	Day 1: 125 mg. ADD. 1 hour prior to chemotherapy	Days 2-3: 80 mg	≤ 3 days
	Fosaprepitant - Ampoule (injection): 150 mg	IV dose: 150 mg IV SD*	IV dose: 150 mg IV SD*	IV dose: 150 mg IV SD*	SD administration: Preferably 30 minutes prior to chemotherapy

Sequential Therapy and Stepwise Management Algorithms

In patients with cancer who present with nausea and vomiting, once reversible causes are addressed, it is crucial to establish a pharmacological plan that maintains ongoing symptom relief while avoiding unnecessary exposure to multiple agents [3,22]. In many cases, monotherapy or initial dual combinations are adequate; however, in refractory or prolonged cases, a sequential approach may be needed to optimize clinical response and reduce cumulative toxicity [22,39].

Unlike chemotherapy- or radiotherapy-induced emesis, for which international consensus guidelines and well-established algorithms exist, choosing and sequencing antiemetics in other oncological situations remains difficult, especially when multiple underlying mechanisms are involved [5,12]. The proposed model is based on the main pathophysiological mechanism, the available evidence for each drug class, and considerations of accessibility and tailoring treatment to the individual patient's clinical profile [35,39,60].

Monotherapy is appropriate in mild cases or when a single pathophysiological mechanism dominates, as supported by most clinical guidelines [2,5,15]. However, in cases of multifactorial nausea and vomiting, combination therapy targeting different receptor pathways has shown improved symptom control, especially in palliative care settings, although strong evidence for its routine initial use outside of chemotherapy remains limited [12,15].

Management may begin with ondansetron as the first-line therapy, in accordance with international recommendations [36,41,56]. Alternatively, metoclopramide can be started in patients who are intolerant to ondansetron or have concurrent constipation, which is common in this population [41,56]. If the clinical response is satisfactory, the regimen should be maintained for the shortest effective duration, with daily reassessment [12,17]. Although there is no consensus on the optimal treatment duration, full-dose therapy should generally not exceed seven days, followed by a gradual taper for at least three days to prevent symptom rebound [35,39]. When the response is partial but clinically acceptable, a second agent should be added after 48 hours before escalating to maximum doses; if this is insufficient, full titration may then proceed [12,35].

If the response to ondansetron monotherapy remains incomplete after 48-72 hours, a second agent with a different mechanism of action should be introduced, such as metoclopramide if not previously used [17,18,61]. This agent may be titrated to the maximum recommended dose over a similar timeframe, with a total treatment duration of no more than seven days [17,18,61]. When sustained serotonergic modulation is needed, oral mirtazapine may be considered when feasible; although supporting evidence remains limited, it may be especially helpful for patients with concurrent insomnia, anxiety, or anorexia [58,59]. If continued dopaminergic antagonism is necessary, oral olanzapine or parenteral alizapride may be used, with gradual dose adjustments based on response and tolerability [19,22,60,61].

Therapeutic failure is defined as persistent nausea with an intensity of ≥4 on a 0-10 numerical rating scale or ≥2 vomiting episodes within 24 hours, despite adequate use of at least two antiemetic regimens with different mechanisms of action. This should be accompanied by functional impairment or sustained intolerance to oral intake [35,36]. If this scenario is confirmed between days four and six of treatment, after reaching maximum doses of ondansetron and metoclopramide, third-line therapy with corticosteroids, primarily dexamethasone, should be considered for two to five days [22,35,62]. At this stage, early reassessment (≤8 hours) is essential to confirm adherence, exclude untreated causes such as bowel obstruction, hypercalcemia, or brain metastases, and identify adverse effects requiring modification [12,44]. Clinical response should be reassessed every 24 hours, with sequential adjustments every 48-72 hours if improvement is not observed, and agents with different mechanisms of action should be consistently prioritized [17,43].

Interdisciplinary Consensus-Based Management Strategy

**Figure 3:**
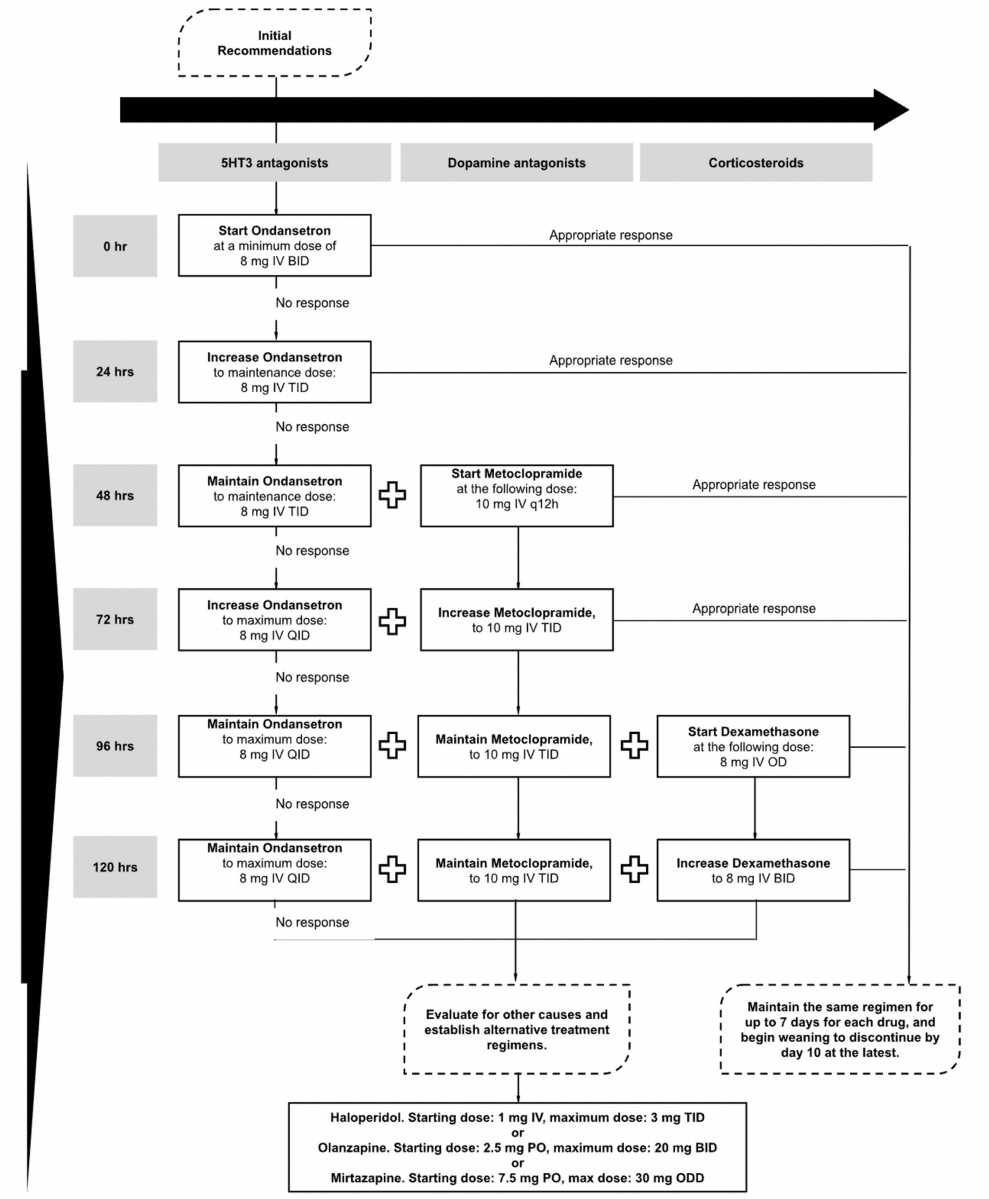
Stepwise sequential management of opioid-induced nausea and vomiting in patients with cancer. ODD: once daily; BID: twice daily; TID: three times daily; QID: four times daily. Source: Developed by the authors using Microsoft Word (Microsoft Corporation, Redmond, WA) based on references [1-7].

Figure 3 presents a clinical algorithm for the management of OINV in oncology patients receiving multimodal opioid-based analgesia, serving as a practical decision-support tool in high-complexity clinical settings.

Indications for Specialist Referral

Persistent nausea or vomiting despite properly implemented and reassessed stepwise pharmacological management clearly indicates the need for referral to specialized care, especially when there is suspicion or confirmation of mechanical bowel obstruction, peritoneal carcinomatosis, intestinal pseudo-obstruction, or progression of abdominal tumors [36]. In such cases, an interdisciplinary evaluation is often necessary, considering advanced supportive measures such as nasogastric or percutaneous decompression, venting gastrostomy in cases of distal non-resolvable obstruction, or interventional techniques such as celiac or splanchnic plexus blocks to manage complex visceral symptoms [39]. Early referral helps optimize symptom control, prevents prolonged therapeutic failure, and reduces the risk of unnecessary or excessive interventions in patients with advanced disease [39].

Future directions

Future guidelines for managing nausea and vomiting in oncology should shift toward a personalized, predictive approach, supported by validated clinical predictors and risk-stratification tools, such as recently developed prognostic models for chemotherapy-induced emesis [17,38]. At the same time, pharmacogenetics should be gradually integrated into clinical decision-making, given the well-documented individual variability in antiemetic response associated with relevant genetic polymorphisms [11,38]. Incorporating pharmacogenomic insights may improve treatment precision and reduce unnecessary drug exposure in vulnerable groups. It is equally important to enhance adherence to existing clinical guidelines by embedding them into electronic decision-support systems, structured continuing medical education programs, and coordinated multidisciplinary care pathways [11,36]. Finally, future research should focus on clinical scenarios that remain inadequately addressed, including non-acute emesis, multi-day chemotherapy protocols, and OINV outside the chemotherapy setting. There is also an urgent need to develop therapeutic combinations that offer high clinical efficacy while being affordable and having a positive impact on patient quality of life, all while maintaining healthcare system sustainability [36,38,63].

## Conclusions

Nausea and vomiting in patients with cancer remain frequent, burdensome, and often under-recognized symptoms that significantly impair quality of life, functional status, nutritional health, and adherence to essential therapies, including opioid analgesia. Their multifactorial pathophysiology requires a structured diagnostic approach to identify the primary mechanism and guide rational, mechanism-based treatments.

This manuscript presents a practical, step-by-step clinical framework applicable in various care settings, including those with limited resources. By combining systematic evaluation of causes, targeted opioid risk assessment, sequential antiemetic strategies, and clear referral criteria, the proposed model aims to improve symptom control while reducing unnecessary polypharmacy and therapy escalation. Ongoing reassessment and interdisciplinary collaboration are emphasized as vital components of safe and effective care, especially in difficult or complex cases. Importantly, this approach considers real-world healthcare limitations while encouraging evidence-based, proportionate clinical decision-making. By offering practical tools for evaluation, prevention, and management, it helps reduce unnecessary variation in clinical practice and promotes more consistent, patient-centered care throughout the cancer journey. Ultimately, comprehensive and individualized symptom management is fundamental to high-quality palliative oncology and a key factor in maintaining dignity in advanced disease.
